# Discovering functional impacts of miRNAs in cancers using a causal deep learning model

**DOI:** 10.1186/s12920-018-0432-0

**Published:** 2018-12-31

**Authors:** Lujia Chen, Xinghua Lu

**Affiliations:** 1 Department of Biomedical Informatics, School of Medicine, University of Pittsburgh, 5607 Baum Blvd, Pittsburgh, PA USA; 2 Center for Causal Discovery, University of Pittsburgh, 5607 Baum Blvd, Pittsburgh, PA USA; 3 Department of Pharmaceutical Sciences, School of Pharmacy, University of Pittsburgh, 5607 Baum Blvd, Pittsburgh, PA USA

**Keywords:** Deep learning, Causal discovery, miRNA and mRNA

## Abstract

**Background:**

Micro-RNAs (miRNAs) play a significant role in regulating gene expression under physiological and pathological conditions such as cancers. However, it remains a challenging problem to discover the target messenger RNAs (mRNAs) of a miRNA in a data driven fashion. On one hand, sequence-based methods for predicting miRNA targets tend to make too many false positive calls. On the other hand, analyzing expression correlation between miRNAs and mRNAs cannot establish whether relationship between a pair of correlated miRNA and mRNA is causal.

**Methods:**

In this study, we designed a deep learning model, referred to as miRNA causal deep net (mCADET), which aims to explicitly represent two types of statistical relationships between miRNAs and mRNAs: correlation resulting from confounded co-regulation and correlation as a result of causal regulation. The model utilizes a deep neural network to simulate transcription mechanism that leads to co-expression of miRNA and mRNA, and, in addition, it also contains directed edges from miRNAs to mRNAs to capture causal relationships among them.

**Results:**

We trained the mCADET model using pan-cancer miRNA and mRNA data from The Cancer Genome Atlas (TCGA) project to investigate mechanism of co-expression and causal interactions between miRNAs and mRNAs. Quantitative analyses of the results indicate that the mCADET significantly outperforms conventional deep learning models when modeling combined miRNA and mRNA expression data, indicating its superior capability of capturing the high-order statistical structures in the data. Qualitative analysis of predicted targets of miRNAs indicate that predictions by mCADET agree well with existing knowledge. Finally, the predictions by mCADET have a significantly lower false discovery rate and better overall accuracy in comparison to sequence-based and correlation-based methods when comparing to experimental results.

**Conclusion:**

The mCADET model can simultaneously infer the states of cellular signaling system regulating co-expression of miRNAs and mRNAs, while capturing their causal relationships in a data-driven fashion.

## Background

### Regulated expression of miRNAs and their regulatory functions

Micro-RNAs are a class of small RNAs, about 22 nucleotides in length and involved in post-transcriptional and translation regulation of gene-expression either by direct cleavage of mRNA or translational repression [1]. In the last decade, studies show that the dysfunction/dysregulation of certain miRNAs are involved in the development and progression of many diseases. Particularly, the role of miRNA in cancer has drawn attention in last decade. Studies demonstrated that the dysregulation of miRNA expression could lead to human cancers [2]. The mechanisms include amplification or deletion of miRNA genes [3], abnormal transcriptional control of miRNAs [4], dysregulated epigenetic changes [5, 6] and deficiencies in the miRNA biogenesis machinery [7]. The loss of tumor suppressor miRNAs or overexpression of oncogenic miRNAs can lead to breast cancer tumorigenesis or metastasis [8, 9].

To gain a better understanding of the roles of miRNAs in normal biological processes and in the development of disease, it is important to accurately identify which genes are targeted by each miRNA. Since it is infeasible to perform biological experiments for such a large number of miRNAs, computational methods play an important role in studying miRNA, and numerous computational methods have been developed for predicting targets of miRNAs. To predict the interaction between miRNA and mRNA, many tools have been developed using different algorithms [10–14], although two main approaches dominate the field. One approach is the sequence-based miRNA target prediction, which models the complementary sequence similarity between miRNA and mRNA and structural stability of the putative duplex to predict whether a mRNA is a target of a miRNA [12, 15–17]. Give a miRNA sequence dataset (e.g., miRBase [18], StarBase [11]), sequence-based models can be used to scan whole mRNA transcriptome to predict potential targets. However, these methods have been shown to have a high rate of false positives and false negatives [19]. This is mainly because sequence similarity is not sufficient to predict the folding of RNA duplexes and their interaction with the proteins involved in miRNA-mediated regulation [19, 20].

Another common approach of studying miRNA and mRNA relationship is the correlation-based miRNA target prediction. Based on miRNA and mRNA expression data collected from a cohort of biological samples, correlation-based methods search for anti-correlation relationships between pairs of miRNA and mRNA as potential regulator-target pairs. Different databases based on correlation analyses are available, e.g., mirCox [21]. However, an anti-correlation between a pair of miRNA and mRNA does not necessarily represent a causal relationship. It is not uncommon that a signaling pathway may simultaneously regulate expression of a miRNA and a set of mRNAs, which may lead to confounded correlation. As it is often said: *correlation does not entail causality*. Thus more rigorous causal inference methods are needed to infer the causal relationships between miRNA and mRNA.

In this study, we present a novel method of studying the statistical relationships between mRNAs and miRNAs by analyzing large-scale data from TCGA. Our method integrates two complementary machine learning frameworks: deep learning and causal inference. Our model, referred to as miRNA causal deep net (mCADET), consists of deep neural network that can capture the transcriptomic machine that controls expression of both miRNA and mRNAs to capture the statistical structure resulting from co-regulation, and, in addition, it includes directed edges from miRNA to mRNA to capture the potential causal relationships between miRNA and mRNA. We show that this integrative approach can significantly outperform the sequence-based and correlation-based methods in predicting targets of miRNAs.

## Methods

### Data collection

The miRNA and mRNA expression data were obtained and downloaded from TCGA consortium website (https://cancergenome.nih.gov/). For the breast cancer (BRCA), 1218 mRNA sequencing samples were downloaded with 20,531 mRNAs, and 1701 miRNA sequencing samples were downloaded with 1918 microRNAs, which includes duplicate and triplicate samples. We further identified tumor samples with both mRNA and miRNA measurements. We discretized (binary) the expression data by comparing the expression values of mRNA and miRNAs from tumor samples with those from normal samples profiled by TCGA using the 3-fold change. Finally, we merged the miRNA and mRNA dataset into a 757 ×22,449 (samples × combined miRNA and mRNA) binary matrix for model training.

Several open-resource miRNA-target databases are used in this paper including miRTarBase (an experimentally validated database) [22], and miRDB (a sequence based prediction database and the prediction tool used is MirTarget V3) [23, 24]. TMREC [25] and TTRUST [26] were used to look up the TF-miRNA and TF-mRNA interactions separately to help find the common TF regulating both a particular miRNA and a particular mRNA.

### Model

#### Model architecture

**Fig. 1:**
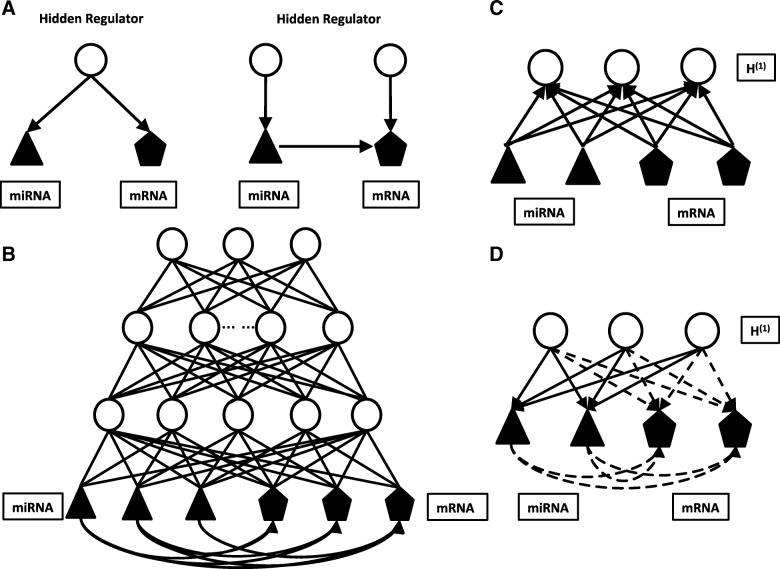
Illustration of the mCADET model. **a** An example of the causal relationship between high correlated miRNA-mRNA pairs. The triangles represent miRNAs. The pentagons represent mRNAs and the circles represent hidden regulators. **b** mCADET model with edges added from observed miRNA (visible units) to observed mRNA (visible units) to reflect the regulatory role of miRNA on the expression of mRNA. **c** Semi-RBM positive phase **d** Semi-RBM negative phase. Solid lines represent the update for miRNAs and dotted lines represent the update for mRNAs

As we mentioned above, the high correlation doesn’t guarantee the causality. The correlation between miRNA and mRNA could be a result of two types of regulations: 1) Transcriptions of a miRNA and a mRNA are regulated by a common cellular signal (Fig. 1a), which can be a common pathway or a common transcription factor (TF). In other words, expressions of the miRNA and mRNA are confounded by a common latent variable. 2) Expression of a miRNA and a mRNA are regulated by distinct cellular signals, but miRNA can causally regulate the degradation of mRNA (Fig. 1a). To capture these two types relationships, we designed a hybrid model, which uses a deep autoencoder to capture the signals of cellular signaling systems to explain the coregulation of miRNAs and mRNAs and further includes causal edges from miRNAs to mRNAs to capture the causal relationships. The regulatory type between miRNAs and mRNAs could be reflected by +/− sign of the predicted interaction value.

An autoencoder uses multiple layers of latent variables (hidden nodes) to capture compositional statistical structures in a distributed manner, such that each layer captures the structure of different degrees of abstraction. As shown in Fig. 1b, an autoencoder contains one visible (input) layer and one or more hidden layers. To efficiently train the autoencoder, we treat it as a series of two-layered restricted Boltzmann machines (RBM) stacked on top of each other [27]. The inference of the states of hidden node and learning of model parameters are performed by learning the RBM stacks in a bottom-up fashion, which is followed by a global optimization of generative parameters using the back-propagation algorithm [28]. As shown in our previous studies [29, 30], autoencoders are capable of capturing signals regulating gene expressions under different settings. For example, the first hidden layer could represent the transcription factors (TFs).

To further capture the causal relationships between miRNAs and mRNAs, we designed a deep belief network (DBN) model containing directed edges from miRNA to mRNAs. We pre-train the model by treating the observed variables (miRNA and mRNA) and first hidden layer above them as a semi-RBM. The semi-RBM between the first two layers allows the edges from miRNA visible nodes to mRNA visible nodes to reflect the regulatory role of miRNA in gene expression. The layers above the second layer still use the traditional RBMs as shown in Fig. 1b. The followings show how the positive phase and negative phase perform. Under this setting, when the correlation between a pair of miRNA and mRNA are competently explained by co-regulation or causal edges, regularization techniques (discussed later) potentially constrain the model to pick one dominant mechanism to represent the correlation.

#### Semi-RBM positive phase

As shown in Fig. 1c, both miRNA and mRNA contribute to the activation of hidden units in the first hidden layer.

$$ \mathrm{P}\left({h}_j|{v}_{\mathrm{mrna}\&\mathrm{mirna}i}=1\right)=\upsigma \left({b}_j+\sum \limits_{\mathrm{i}=1}^{\mathrm{n}}{\mathrm{W}}_{ij}{{\mathrm{v}}_{\mathrm{mrna}\&\mathrm{mirna}}}_i\right) $$

where *v*_mrna & mirna*i*_ represents the binary state of *i*th visible unit of mRNA and miRNA; *h*_*j*_ represents the state of *j*th hidden unit; *b*_*j*_ represents the bias for the *j*th hidden unit; W_*ij*_ represents the weight between the *i*th visible unit of mRNA and miRNA and the *j*th hidden unit.

#### Semi-RBM negative phase

As shown in Fig. 1d, only the hidden units in the first hidden layer contribute to the activation of the mRNA. However, both the hidden units in the first hidden layer and the mRNAs contribute to the activation of miRNAs.

$$ \Pr \left({\mathrm{v}}_{\mathrm{m}\mathrm{irna}\_\mathrm{k}}=1|\mathrm{h}\right)=\upsigma \left({\mathrm{a}}_{\mathrm{m}\mathrm{irna}\_\mathrm{k}}+\sum \limits_{\mathrm{j}=1}^{\mathrm{m}}{\mathrm{W}}_{\mathrm{kj}}{\mathrm{h}}_{\mathrm{j}}\right) $$

$$ \Pr \left({\mathrm{v}}_{\mathrm{m}\mathrm{rna}\_\mathrm{o}}=1|\mathrm{h},{\mathrm{v}}_{\mathrm{m}\mathrm{irna}}\right)=\upsigma \left({\mathrm{a}}_{\mathrm{m}\mathrm{rna}\_\mathrm{o}}+\sum \limits_{\mathrm{j}=1}^{\mathrm{m}}{\mathrm{W}}_{\mathrm{oj}}{\mathrm{h}}_{\mathrm{j}}+\sum \limits_{\mathrm{k}=1}^{\mathrm{p}}{\uppi}_{\mathrm{ok}}{\mathrm{v}}_{\mathrm{m}\mathrm{irna}\_\mathrm{k}}\right) $$

$$ {\mathrm{v}}_{\mathrm{mrna}\&\mathrm{mirna}}=\mathrm{cbind}\left({\mathrm{v}}_{\mathrm{mrna}},{\mathrm{v}}_{\mathrm{mirna}}\right) $$

where v_mirna _ k_ represents the *k*th visible unit of miRNA; W_kj_ represents the weight between the *k*th visible unit of miRNA and the *j*th hidden unit; a_mirna _ k_ represents the bias for the *k*th visible unit of miRNA; v_mrna _ o_ represents the *o*th visible unit of mRNA; a_mrna _ o_ represents the bias for the *o*th visible unit of mRNA; W_oj_ represents the weight between *o*th visible unit of mRNA and the *j*th hidden unit; π_ok_ represents the weight between *o*th visible unit of mRNA and the *k*th visible unit of miRNA. cbind represents the combination of mRNA and miRNA for the same sample.

#### Semi-RBM parameter update

$$ \Delta  {\mathrm{w}}_{\mathrm{ij}}=\upepsilon \left(<{\mathrm{v}}_{\mathrm{mrna}\&\mathrm{mirna}\_\mathrm{i}}{\mathrm{h}}_{\mathrm{j}}{>}_{\mathrm{data}}-<{\mathrm{v}}_{\mathrm{mrna}\&\mathrm{mirna}\_\mathrm{i}}{\mathrm{h}}_{\mathrm{j}}{>}_{\mathrm{model}}\right) $$

$$ \Delta  {\uppi}_{\mathrm{ok}}=\upepsilon \left(<{\mathrm{v}}_{\mathrm{mrna}\_\mathrm{o}}{\mathrm{v}}_{\mathrm{mirna}\_\mathrm{k}}{>}_{\mathrm{data}}-<{\mathrm{v}}_{\mathrm{mrna}\_\mathrm{o}}{\mathrm{v}}_{\mathrm{mirna}\_\mathrm{k}}{>}_{\mathrm{model}}\right) $$

More details of the algorithm and pseudo code for training a traditional autoencoder were discussed in both literature and our previous work [29, 30]. The backpropagation process is the same as the standard one except that we update the visible units of miRNA using the hidden units in the first hidden layer only, but use both hidden units in the first hidden layer and the visible units of miRNA to update the visible units of mRNA.

#### Regularization

The number of miRNA and mRNA training samples is limited compared with the dimension of miRNA and mRNA features. Therefore, we applied the techniques of regularization to the first two hidden layers to reduce the risk of overfitting. When we train a traditional RBM model, each hidden unit is fully connected to each observed unit and a non-zero weight is usually assigned to each pair of observed unit and hidden unit. However, in the real cases of cellular regulatory mechanism, the change in mRNA and miRNA expression is often induced by a small number of biological components such as TFs or pathways. This enables one to specify that only a certain percent of hidden units have a high probability to be set to 1 (“on”) by adding a penalization term to the optimization function. In this model, regularization was only added to the first hidden layer [31]. During the RBM training within an autoencoder, the optimization of the sparse RBM minimizes the negative log-likelihood of the data with the addition of the regularization term [32].

$$ {\operatorname{minimize}}_{\left[\theta \right]}-{\sum}_{l=1}^{\mathrm{s}}\log {\sum}_{j=1}^{\mathrm{n}}\mathit{\Pr}\left({\mathrm{v}}^l,{\mathrm{h}}_j^l|\theta \right)+\uplambda {\sum}_{j=1}^{\mathrm{n}}\mid p-\frac{1}{\mathrm{s}}{\sum}_{l=1}^{\mathrm{s}}\mathrm{E}\Big[{\mathrm{h}}_j^l{\left|{\mathrm{v}}^l\Big]\right|}^2 $$

where θ is the parameter vector [a, b, W]; s is the total number of samples; n is the total number of hidden units; λ is the regularization constant threshold and *p* is a constant controlling the percent of hidden units h_*j*_ to be active (the sparseness of the hidden units h_*j*_). Effective model selection was performed to select the best sparsity threshold leading to the lowest cost function.

#### Model training

We trained models under several configurations. We trained a deep belief network (DBN) model without edges from miRNAs to mRNAs, the mCADET model with edges from miRNAs to mRNAs but without regularization, and the mCADET model with edges from miRNAs to mRNAs and with regularization (different sparsity ratios), and finally a the mCADET model with random permutation of miRNAs and mRNAs across tumors where the statistical relationships between miRNAs and mRNAs were fully disrupted. We compared the reconstruction errors for these models to choose the best model. The reconstruction errors are the difference between the raw input data and the reconstructed input predicted from the model. The model with the best reconstruction error is chosen to conduct the evaluations of biological representations.

### Evaluations

We used the experimental results from miRTarBase [22] as “gold standard” to evaluate the prediction by our models and those by sequence-based and correlation-based methods. The accuracies of regulatory miRNA-mRNA pairs predicted by mCADET models were compared with ones predicted by correlation-based and sequence-based analysis separately. For the correlation analysis, we run pair-wise linear regression on our dataset to identify pairs with statistically significance, corrected by false discovery. For the sequence-based analysis, we used the predicted miRNA-mRNA interaction acquired from the miRDB database [23]. Since it is difficult to assess the true negative rate, we mainly concentrate on evaluating models’ capabilities in identifying the true positives, i.e., the recall and positive predictive value (PPV) of each method.

$$ recall=\frac{TP}{TP+ FN}, $$

$$ PPV=\frac{TP}{TP+ FP}. $$

To test whether the PPVs for two methods are significantly different from each other, we calculate the z-score as follows,

$$ \mathrm{z}=\frac{\widehat{{\mathrm{p}}_1}-\widehat{{\mathrm{p}}_2}}{\sqrt{\widehat{\mathrm{p}}\left(1-\widehat{\mathrm{p}}\right)\left(\frac{1}{{\mathrm{n}}_1}+\frac{1}{{\mathrm{n}}_2}\right)}}, $$

$$ \widehat{p}=\frac{{\mathrm{n}}_1\widehat{{\mathrm{p}}_1}+{\mathrm{n}}_2\widehat{{\mathrm{p}}_2}}{n_1+{n}_2}, $$

where *p* represents the PPV for mCADET-based analysis, correlation-based analysis and sequence-based analysis separately; *n* represents the number of predicted mRNAs. The *z* value between mCADET versus sequence-based model and the *z* value between mCADET versus correlation-based analysis were calculated separately. To quantitatively evaluate the difference between precision and recall for two groups, we calculated the F1 score that evaluates weighted average of precision and recall [33].

$$ \mathrm{F}1\ \mathrm{Score}=\frac{2\ast \left(\mathrm{Recall}\ast \mathrm{Precision}\right)}{\left(\mathrm{Recall}+\mathrm{Precision}\right)} $$

Finally, we also validated predicted mRNA-miRNA pairs by checking the agreement with literatures.

## Results

### Model training

**Fig. 2:**
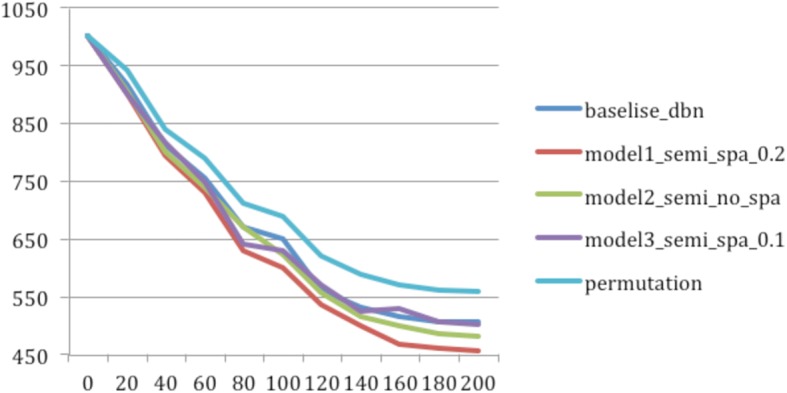
The reconstruction errors between real and reconstructed input for five models of different architectures. The y axis is the reconstruction error and the x axis is the number of epochs. The dark blue, red, green, purple and light blue lines represent the baseline deep belief network, mCADET with sparsity ratio 0.2, mCADET without regularization, mCADET with sparsity ratio 0.1 and mCADET with data permutation separately

We first assessed the capability of models to capture and represent statistical structures of data by comparing the reconstruction errors of different models in a series of cross-validation experiments. We compared different DBN models with a fixed model architecture with four hidden layers and each layer has 1500, 1000, 500, and 250 nodes respectively. The baseline DBN is a DBN model without edges from miRNA to mRNA; the Model1_semi_spa_0.2 is a mCADET model that allows edges from miRNA to mRNA (i.e., the observed and first hidden layer forms a semi-RBM) with a sparsity threshold 0.2; Model3_semi_spa_0.1 is an mCADET model with edges from miRNA to mRNA with a sparsity threshold 0.1. This means that we added a penalty to the activation of hidden units to allow 10% of the hidden units to be active. The result shows that the model with edges from miRNA to mRNA and with a sparsity threshold 0.2 has the lowest reconstruction error (Fig. 2 and Table 1). The result (Table 1) shows that the model allowing edges with a sparsity threshold 0.2 has the lowest reconstruction error. Therefore, the result analysis from the perspective of biological knowledge showed below is all based on this model.

**Table 1:** The reconstruction errors for experimentally validated miRNA-targets only

Models	miR374a (414 targets)	miR15b (265 targets)	miR190 (428 targets)
Baseline_dbn	55.78	48.64	74.26
Model1_semi_spa_0.2	**50.46**	**43.66**	**64.74**
Model2_semi_no_spa	52.18	44.81	68.46
Model3_semi_spa_0.1	53.28	45.23	65.35
Permutation	61.23	67.25	95.45

The following interesting observations are noteworthy: 1) Adding causal edges between miRNA and mRNA improves the capability of a model to capture the overall statistical structures of data. Indicating that these potential causal edges enabled the model to capture the statistical relationships between miRNA and mRNAs that cannot be fully explained by co-regulation. 2) Right amount of regularization enhances the capability of model to capture the statistical structures. This is reflected by the fact that model1_semi-spa_0.2 outperforms models without regularization (model2_semi_no_spa) and the model with too few parameters (model3_semi_spa_0.1 that only allow 10% of the hidden nodes to be active).

### Statistical evaluation of predicted miRNA-mRNA interaction

In the deep learning model, the weights of the direct edges from miRNAs to mRNAs reflect the strength of interaction between miRNA and mRNA. Therefore, we used the weights trained by the deep learning models to perform the miRNA-mRNA interaction analysis. We only keep the top 5% absolute weights for each miRNA and get a corresponding mRNA list.

**Fig. 3:**
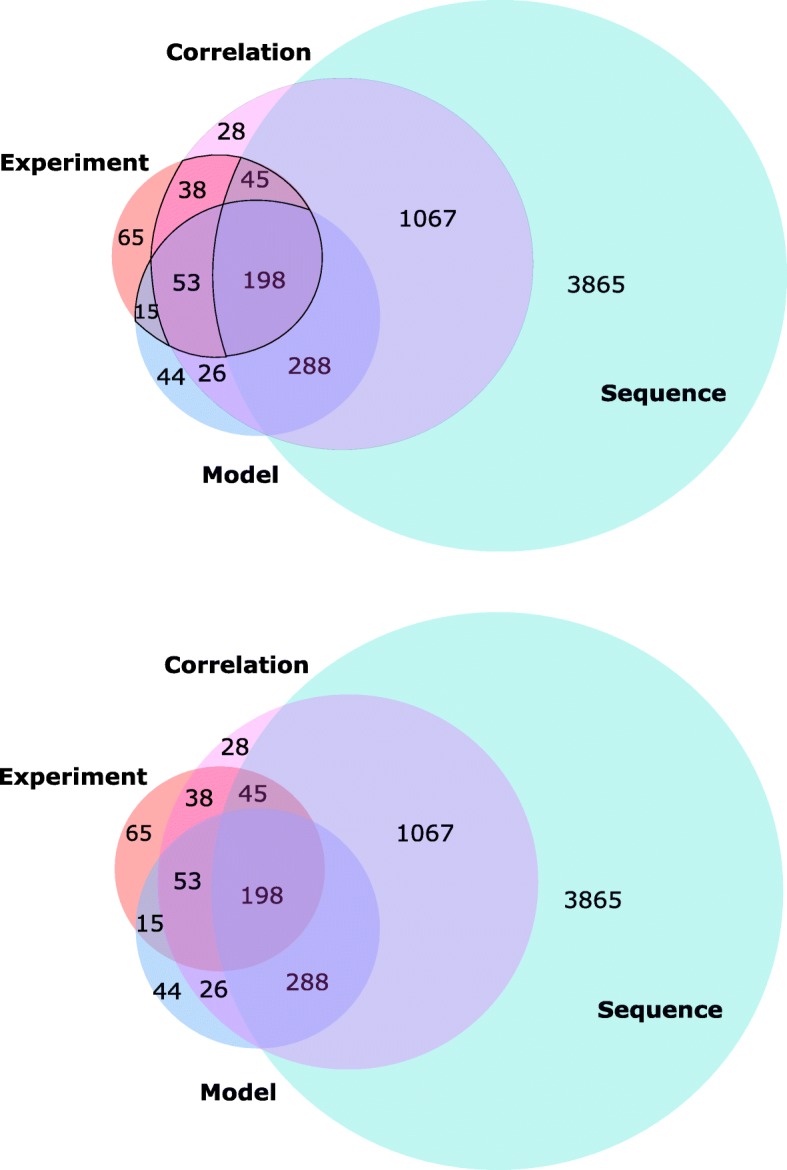
The Venn diagram of predicted targets of miR-374a for mCADET-based, correlation-based, sequence-based and experimentally validated targets separately. The blue circle is the mCADET-based analysis. The purple circle is the correlation-based analysis. The light green circle is the sequence-based analysis and the orange circle is the experimentally validated mRNA targets

We compared the interactions predicted by the mCADET with the ones predicted by the correlation-analysis and the sequence-based analysis. As a concrete example, we showed the results of different methods predicting the targets of miR-374a, as shown in Fig. 3. Apparently, sequenc-based methods predicted the largest number of potential targets and very few of them match experimental results (PPV ~ 6%). In other words, majority of the predicted targets by sequence-based approach is false positive. Compared with the sequence-based method, the correlation analysis returns less targets. It only finds high correlations between the expression of mRNAs and its regulating miRNAs. In this case, it found 2350 mRNAs with relatively high correlation scores with miR-374a, which means that those mRNAs have higher probability of interacting with miR-374a.

Compared both sequence-based and correlation-based analyses, the number of targets predicted by the mCADET is the smallest. However, the mCADET achieves the highest PPV and best overall performance in comparison to the other two approaches. By comparing the overlap rates between the predicted targets and the experimentally validated targets, the mCADET performs the best as shown in Fig. 3 and Table. 2. In the light that computational predictions eventually need to be experimentally validated, a high PPV is a very desirable characteristics for prediction models. If one interprets the results from Table 2 literally, close to half (43%) of predicted target potentially can be verified by experiments, whereas only 6 and 19% of predictions by sequence-based and correlation-based can be verified. For example, mRNA MTPN and WWP1 are false predicted by sequence-based analysis, however, mCADET gave the interaction a small weight.

**Table 2:** The sensitivity, positive predictive value (PPV) and F1 score of mCADET-based, sequence-based and correlation-based prediction for miRNA-374a separately

miR-374a	Sensitivity (recall)	PPV (precision)	F1
mCADET	0.64	***0.43***	0.51
Sequence- based	0.59	0.06	0.11
Correlation-based	***0.76***	0.19	0.30

We used z-score to test the significant difference of PPVs for each two analyses. After the calculation, the *z* value and *p*-value between model-based analysis and correlation-based analysis is 2.67 and 0.045 respectively. The *z* value and p-value between model-based analysis and sequence-based analysis is 3.25 and 0.038 respectively. Both of *z* scores are bigger than 1.96 [34] and *p*-values are less than 0.05, which shows that each of two groups is significantly different from each other. We could also conclude from Table 2 that the performance of our mCADET model is better than sequenced-based and correlation-based model. Besides, the sensitivity of the baseline DBN is 0.61 compared with the mCADET 0.64 and the PPV of the baseline DBN is 0.28 compared with the mCADET 0.43.

### mCADET provides insights of different mechanisms for correlation between miRNAs and mRNAs

As shown in previous sections, adding causal edges in mCADET model can enhance the capability of the model to capture the overall statistical.

Structures including both miRNAs and mRNAs. Comparison to experimentally validated targets indicates that mCADET is more likely to capture the causal relationships between miRNAs and mRNAs. We further examined the examples that strong correlations between miRNAs and mRNAs are explained by different mechanisms in the results learned from the mCADET model.

**Fig. 4:**
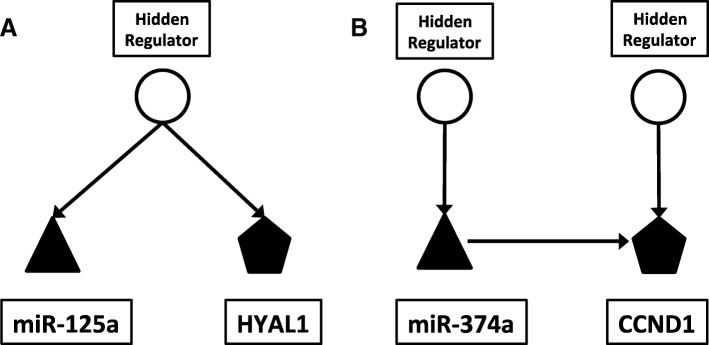
An example of the causality among hidden regulator, miRNA and mRNA inferred from mCADET. The triangles represent miRNAs. The pentagons represent mRNAs and the circles represent hidden regulators. **a** The miR-125a and HYAL1 are co-regulated by a hidden regulator. **b** A causal edge was found from miR-374a to CCND1 with separate hidden regulators

Correlation analysis assigns miR-125a and *HYAL1* pair a strong correlation value 0.78 with a *p*-value 0.03 and, in the mCADET model, the two RNAs are strongly connected to a common hidden node and the candidate causal edge between them does not pass our selection threshold. In other words, the model detected that the two RNAs were regulated by a hidden regulator (potentially a common transcription factor), and there was no strong causal relationship between the two RNAs as shown in Fig. 4a. On the other hand, miR-374a and *CCND1* pair also shows a strong correlation, and mCADET detected a strong direct edge from miR-374a to *CCND1* (as shown in Fig. 4b)*,* indicating a direct regulatory mechanism which is supported by literature [35].

### Interaction network of miRNA-targets

**Fig. 5:**
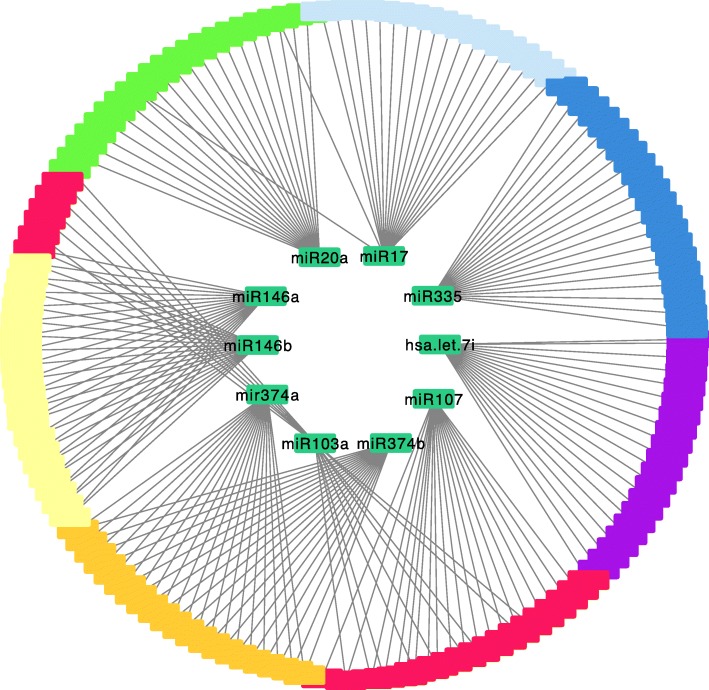
Example of the interaction network of miRNA and mRNA in breast cancer tumors. The interactions between top 10 breast cancer related miRNAs and their top 20 mRNA targets were plotted using the Cytoscape. The inner circle represented by green blocks is the miRNAs and the outer circle represented by ten different colors is the top 20 mRNAs regulated by each miRNA respectively

It is quite common that certain miRNAs share target mRNAs and form a miRNA regulatory network. To test whether the results of the mCADET can be used to search for such networks. We identified top 20 mRNAs associated with a miRNA and organized the miRNAs and mRNAs in a plot shown in Fig. 5. Interestingly, our methods correctly identified members of miRNA families (e.g., miR-146 s and miR-374 s) sharing target mRNAs in a pure data-driven fashion, without utilizing any knowledge of sequence similarity among the members of the miRNA family [36]. This provides additional evidence that our model can correctly detect the causal relationships from distinct miRNAs and to a common set of target mRNAs.

In addition to detect common targets of a miRNA family, our model can also detect the common functional impacts of distinct miRNAs. For example, our model can detect that *CCND1* is the shared target of miR-17 and miR-20a [37]. More such relationships can be found in a broader analysis of our results, which are not shown in Fig. 5.

### Literature-based evaluation of predicted miRNA-mRNA interactions

Previous research has accumulated a rich body of knowledge of regulatory relationships between miRNAs and important cancer drivers. We searched our results to identify predicted regulator miRNAs for certain common cancer driver genes. Many of them are reported in the literature. Table 3 lists examples of predicted mRNA-miRNA pairs validated by literatures.

**Table 3:** Examples of predicted miRNA-mRNA pairs validated by literature

miRNAs	Targes	Function	Reference
miR-146a/miR-146b	*EGFR*	Invasion and metastasis	**MiR**-**146a** suppresses tumor growth and progression by targeting **EGFR** pathway and in ap-ERK-dependent manner in castration-resistant prostate cancer **MiR**-**146b**-5p suppresses **EGFR** expression and reduces in vitro migration and invasion of glioma
miR-335	*SOX4*	Metastasis progression	**miR**-**335** orchestrates cell proliferation, migration and differentiation in human mesenchymal stem cells
miR-17	*CCND1*	Cell cycle, cellular proliferation	The **miR**-**17**-5p microRNA is a key regulator of the G1/S phase cell cycle transition
miR-20a	*CCND1*	Cell cycle, cellular proliferation	MicroRNAs MiR-17, **MiR**-**20a**, and MiR-106b act in concert to modulate E2F activity on cell cycle arrest during neuronal lineage differentiation of USSC
miR-374a/miR-374b	*WNT*	Cell metastasis	**MicroRNA-374a** activates **Wnt**/ß-catenin signaling to promote breast cancer metastasis **MicroRNA-374b** Suppresses Proliferation and Promotes Apoptosis in T-cell Lymphoblastic Lymphoma by Repressing AKT1 and **Wnt-16**
miR-374a/miR-374b	*PTEN*	Cellular proliferation, survival and growth	**MicroRNA-374a** activates Wnt/ß-catenin signaling to promote breast cancer metastasis Increased **miR-374b** promotes cell proliferation and the production of aberrant glycosylated lgA1 in B cells of lgA nephropathy

## Discussion

MicroRNAs play a significant role in regulating gene expression under physiological and pathological conditions. In particular, genomic alterations (amplification/deletion) of miRNAs in cancers have significant impacts on cancer development, disease progression, and therapy responses [38–40]. Thus, revealing the functional impacts of miRNAs in cancer will advance cancer biology. As shown in this report, previous methods of identifying targets of miRNAs have significant limitations. By combining deep learning and causal inference, the reported mCADET model ac-hieved significantly identifying targets of miRNAs. Particularly, the improved PPV will convince cancer biologists to carry out validation experiments with much high confidence, thus helping to advance cancer biology.

The reported mCADET model is motivated by biological insights of the data related to miRNAs and mRNAs. It combines the strength of deep learning and causal inference in solving this important biological problem. The superior performance of the model reflects the importance of integrating biological insights with advance machine learning technology. Possible future improvement of the model includes (but not necessarily limited to) combining genomic alteration data to map hidden nodes to concrete biological entities as we did in mining yeast gene expression data [31].

## Conclusions

In this study, we investigated the utility of the mCADET model to simultaneously infer the states of cellular signaling system regulating co-expression of miRNAs and mRNAs, while capturing their causal relationships in a data-driven fashion. This model can be used by miRNA researchers to systematically search for miRNAs that play significant roles in cancers and understand their disease mechanism which, we anticipate, will make significant advances in cancer biology, beyond what are reported here.

## Abbreviations

DBN: Deep belief network
mCADET: miRNA causal deep net
miRNAs: Micro-RNAs
mRNAs: Messenger RNAs
PPV: Positive predictive value
RBM: Restricted boltzmann machines
TCGA: The cancer genome atlas
TF: Transcription factor
